# Case Illustrating the Advantages of Median Lithotomy

**Published:** 1882-05

**Authors:** George E. Brewer


### Clinical Reports.

### Case Illustrating the Advantages of Median Lithotomy.

Reported by George E. Brewer.

Mr. H., of Pennsylvania, aged 36, suffering with symptoms of stone in the bladder, was brought to Dr. Julius F. Miner for treatment. As the case is one of interest, a brief history of it may not be uninstructive. Early in the preceding February, the patient sustained severe injuries, resulting in retention of urine, which necessitated the use of a catheter for its relief. A flexible rubber instrument was used. Attempting its removal, the attending physician was astounded to find that it had separated, about eight inches remaining in the bladder. He immediately suggested the use of a lithotrite for its removal, but finding that considerable deposit had accumulated before an instrument could be procured, he wisely desisted from any such uncertain and embarrassing procedure.

March 29th. About seven weeks after the accident, Dr. Miner, assisted by Drs. Kebler, Cary, Sloan and others, attempted its removal by the median operation for stone.

Ether was administered, after which the patient was placed in the lithotomy position. A grooved staff was then introduced into the urethra, with which the foreign body could now be distinctly felt. An incision about an inch and a half was then made in the raphe of the perineum, about three quarters of an inch above the anus. This divided the skin, superficial fascia, and a thin layer of adipose tissue. Then the rectum, being drawn downward by the forefinger of the left hand, the knife was carried back to the groove of the staff, making an incision through the membranous portion of the urethra, about three-quarters of an inch in length. A ball-pointed probe, which was then passed along the groove of the staff into the bladder, served as a guide to the finger, which, as soon as the staff was removed, was introduced to dilate the parts. In about ten or fifteen minutes the urethra was sufficiently dilated to admit the lithotomy forceps. Five pieces of catheter, completely encrusted with phosphatic deposit, varying in length from one-half to three inches, were removed. The bladder was then thoroughly syringed out, and the patient placed in bed.

Not an unpleasant symptom followed the operation. The slight hæmorrhage, which was present, ceased as soon as the position of the patient was changed.

During the second day the pain, frequent desire to urinate, the constant muco-purulent discharge, and other unpleasant symptoms present before the operation, had nearly disappeared. The patient had good control over his bladder, passing water only once in two or three hours.

April 5th.—One week after the operation. His temperature has never risen above 99½°, nor his pulse exceeded 110. Part of the urine has already passed through the urethra; wound looking healthy and granular.

April 12th.—Two weeks after the operation. The patient in excellent condition; passes nearly all the urine through urethra; wound nearly closed. Every indication of speedy recovery.

The merits of median lithotomy have been variously estimated by different surgeons. While none actually condemn it, very few have much to say in its favor. The reason it is not more commonly practiced at the present day, seems to be owing to the fact that surgeons have not given it a fair trial.

Holmes, speaking of median lithotomy, says that it is "an easy and ready way of removing small stones in *childhood*;" while its application seems to Ashhurst to be "practically limited to those cases among *adults*, in which the stone is small." Erichsen, in comparing the median with the lateral operation, says, "It appears to me that the median operation, when performed in suitable cases, has the advantages over the lateral of being attended with less risk of arterial hæmorrhage, and less injury to the pelvic fascia," (thus avoiding the danger of urinary infiltration); the author then concludes with the statement that, as it can only be used in cases of small stone, it can never take the place of the lateral. Hence we find that the only objection upon which the opponents of median lithotomy seem to unite, is, that its application is limited to cases of small stone.

Previous to the year 1874, Dr. Miner, supposing this objection valid, always employed the lateral operation. During the summer of 1874, however, he succeeded in removing a calculus weighing 587 grains from a boy twelve years of age, by the median method, and since that time has employed it exclusively.

Of the ten operations he has made in this way, every one has been successful, the age of the patients varying from five to seventy-five years, and the size of the stones, from that of a chestnut to that of an ordinary hen's egg. That the wound healed up rapidly, and that the patient had good control over the bladder almost immediately after the operation, were noticeable facts in nearly all the above cases. The reason of this is palpable. As the median operation is founded on the precept "small incision, much dilatation," the knife is only used to a very limited extent.

The median operation also offers an easy and reliable way of practicing lithotrity. By opening the membranous position of the urethra the lithotrite can be used to much greater advantage, and the debris more easily detected and quickly removed, thus avoiding many of the difficulties and dangers attending ordinary lithotrity.

In this connection Dr. Miner suggests, that in case a stone is ever encountered too large to be safely drawn through the dilated urethra, the operator has the choice of two methods; he may either extend the incision, making the medio-lateral operation as practiced by Buchanan, Lee and Gouley, or he may crush the stone and remove the fragments. Although never being obliged to resort to either alternative, in one instance Dr. Miner crushed a very soft stone while removing it with ordinary lithotomy forceps. With the aid of the finger, however, the fragments were so easily located and removed that he recommends it as a safe and ready mode of procedure, when lithotrity seems desirable.

In conclusion, then, its simplicity; the ease with which complications are overcome; its freedom from profuse arterial hæmorrhage; the slight risk of wounding the pelvic fascia, prostate or seminal vesicles, avoiding the danger of urinary infiltration, cellulitis and septicæmia; the rapidity with which the wound closes, and the ease with which cleanliness is maintained during the after-treatment, constitute the reasons why Dr. Miner so earnestly recommends median lithotomy.

